# Neoadjuvant chemoradiation therapy with gemcitabine/cisplatin and surgery versus immediate surgery in resectable pancreatic cancer: Results of the first prospective randomized phase II trial.

**DOI:** 10.1007/s00066-014-0737-7

**Published:** 2014-09-25

**Authors:** Henriette Golcher, Thomas B. Brunner, Helmut Witzigmann, Lukas Marti, Wolf-Otto Bechstein, Christiane Bruns, Henry Jungnickel, Stefan Schreiber, Gerhard G. Grabenbauer, Thomas Meyer, Susanne Merkel, Rainer Fietkau, Werner Hohenberger

**Affiliations:** 1 Department of Surgery, University Hospital Erlangen, Krankenhausstr. 12, 91054 Erlangen, Germany; 2 Department of Radiation Oncology, University Hospital Erlangen, Erlangen, Germany; 3 Department of Radiation Oncology, University Hospital Freiburg, Freiburg, Germany; 4 Department of Surgery, University Hospital Leipzig, Leipzig, Germany; 5 General Surgery, Hospital Dresden-Friedrichstadt, Dresden, Germany; 6 General Surgery, Hospital of Kanton St. Gallen, St. Gallen, Switzerland; 7 Department of Surgery, University Hospital Frankfurt, Frankfurt/Main, Germany; 8 Department of Surgery – Hospital Campus Großhadern, University Hospital Munich, Munich, Germany; 9 Department of Surgery, University Hospital Magdeburg, Magdeburg, Germany; 10 Department of Radiation Oncology, Hospital Coburg, Coburg, Germany; 11 General Surgery, Hospital Ansbach, Ansbach, Germany

**Keywords:** Adenocarcinoma, Chemoradiation, Pancreas, Surgical procedures, operative, Survival, Adenokarzinom, Radiochemotherapie, Pankreas, Operative chirurgische Verfahren, Überleben

## Abstract

**Background:**

In nonrandomized trials, neoadjuvant treatment was reported to prolong survival in patients with pancreatic cancer. As neoadjuvant chemoradiation is established for the treatment of rectal cancer we examined the value of neoadjuvant chemoradiotherapy in pancreatic cancer in a randomized phase II trial. Radiological staging defining resectability was basic information prior to randomization in contrast to adjuvant therapy trials resting on pathological staging.

**Patients and methods:**

Patients with resectable adenocarcinoma of the pancreatic head were randomized to primary surgery (Arm A) or neoadjuvant chemoradiotherapy followed by surgery (Arm B), which was followed by adjuvant chemotherapy in both arms. A total of 254 patients were required to detect a 4.33-month improvement in median overall survival (mOS).

**Results:**

The trial was stopped after 73 patients; 66 patients were eligible for analysis. Twenty nine of 33 allocated patients received chemoradiotherapy. Radiotherapy was completed in all patients. Chemotherapy was changed in 3 patients due to toxicity. Tumor resection was performed in 23 vs. 19 patients (A vs. B). The R0 resection rate was 48 % (A) and 52 % (B, *P* = 0.81) and (y)pN0 was 30 % (A) vs. 39 % (B, *P* = 0.44), respectively. Postoperative complications were comparable in both groups. mOS was 14.4 vs. 17.4 months (A vs. B; intention-to-treat analysis; *P* = 0.96). After tumor resection, mOS was 18.9 vs. 25.0 months (A vs. B; *P* = 0.79).

**Conclusion:**

This worldwide first randomized trial for neoadjuvant chemoradiotherapy in pancreatic cancer showed that neoadjuvant chemoradiation is safe with respect to toxicity, perioperative morbidity, and mortality. Nevertheless, the trial was terminated early due to slow recruiting and the results were not significant. ISRCTN78805636; NCT00335543.

**Electronic supplementary material:**

The online version of this article (doi: 10.1007/s00066-014-0737-7) contains supplementary material, which is available to authorized users.

## Abstract (de)

**Hintergrund:**

Mehrere nichtrandomisierte Studien zeigten, dass eine neoadjuvante Therapie das Überleben bei Patienten mit Pankreaskarzinom verlängert. Beim lokal fortgeschrittenen Rektumkarzinom gehört die neoadjuvante Radiochemotherapie bereits zum Therapiestandard. Analog wurde der Stellenwert einer Radiochemotherapie beim Pankreaskarzinom in einer randomisierten Phase-II-Studie untersucht. Das prätherapeutische radiologische Staging war Grundlage dieser Studie im Gegensatz zu adjuvanten Therapiestudien, die auf pathohistologischem Staging basieren.

**Patienten und Methoden:**

Patienten mit resektablem Pankreaskopfkarzinom wurden randomisiert in primäre Operation (Arm A) versus neoadjuvante Radiochemotherapie gefolgt von einer Operation (Arm B). Beide Gruppen erhielten eine adjuvante Chemotherapie. Es waren 254 Patienten erforderlich, um eine Verbesserung des medianen Gesamtüberlebens von 4,33 Monaten zu erfassen.

**Ergebnisse:**

Die Studie wurde wegen zögerlicher Rekrutierung nach Einschluss von 73 Patienten beendet. Insgesamt konnten 66 Patienten ausgewertet werden. Die ihnen zugeordnete Radiochemotherapie erhielten 29 von 33 Patienten. Alle Patienten bekamen die vollständige Bestrahlungstherapie. Wegen der Toxizität wurde bei 3 Patienten die Chemotherapie reduziert. Eine Pankreastumorresektion erhielten 23 vs. 19 Patienten (A vs. B). Die R0-Resektionsrate betrug 48 % (A) und 52 % (B, P = 0,81). Bei 30 % (A) versus 39 % (B, *P* = 0,44) der resezierten Patienten waren keine Lymphknotenmetastasen vorhanden. Die postoperativen Komplikationen waren in beiden Gruppen vergleichbar. Das mediane Gesamtüberleben betrug 14,4 vs. 17,4 Monate (A vs. B; „Intention-to-treat“-Analyse; *P* = 0,96). Nach Pankreastumorresektion stieg das Gesamtüberleben auf 18,9 vs. 25,0 Monate (A vs. B; *P* = 0,79).

**Schlussfolgerung:**

Diese weltweit erste randomisierte Studie zur neoadjuvanten Radiochemotherapie beim Pankreaskopfkarzinom war in Bezug auf Toxizität sowie perioperative Morbidität und Mortalität gut durchführbar. Die Ergebnisse sind jedoch nicht signifikant, da diese randomisierte Studie vorzeitig wegen mangelnder Rekrutierung beendet werden musste. ISRCTN78805636; NCT00335543.

Survival rates of patients with pancreatic cancer have improved only marginally during the last 30 years with a 5-year survival rate of only 6 % [1]. In contrast, the prognosis of patients with rectal carcinoma has improved substantially during the same timeframe [2]. This progress was due to standardizing surgical therapy [3] worldwide and by the implementation of multimodal therapy [4–6]. Moreover, in rectal cancer it was found early that a clear circumferential margin is important and that even margins below 1 mm cause a significant increase in the rate of local recurrence [7]. All these measures caused a decline in local recurrence from 50 % to about 10 % and an increase of 5-year survival rates up to more than 50 % worldwide. This progress led to the hypothesis that the much poorer prognosis of ductal adenocarcinoma of the pancreas might be improved in an analogous manner.

Adjuvant therapy has been tested in a series of RCT phase III trials, the most important of these are ESPAC-1, CONKO-001, RTOG 97–04, and ESPAC-3 [8–11]. But these trials were still running or results were not yet available when the present trial was planned and conducted. The results of these trials led to a change in standard treatment recommending adjuvant treatment with chemotherapy since 2007 in Germany [12].

The concept of neoadjuvant rather than adjuvant treatment in pancreatic cancer appears attractive for several reasons. First, up to 30 % of the tumors staged as resectable cannot be resected due to undetected metastatic disease or underestimated tumor contact to peripancreatic vessels [13]. Second, up to 30 % of the patients cannot receive adjuvant therapy because of poor post-operative performance status [14]. Both groups of patients are not included into adjuvant trials, though improving overall survival in both arms (adjuvant therapy vs. no adjuvant therapy) by simple patient selection. Neoadjuvant treatment is thought to be better tolerated than adjuvant treatment and avoids postsurgical morbidity in patients with rapidly metastasizing tumors. Nonrandomized trials using the neoadjuvant approach support this rationale: median OS beyond 30 months for patients after neoadjuvant treatment and tumor resection were described in several retrospective data analyses [15–18].

Therefore, in 1999 we started to plan this multicenter randomized phase II study in patients with locally resectable cancer or probably locally resectable cancer of the pancreatic head with strict imaging eligibility criteria defining vascular involvement. To our knowledge, this is the first RCT for patients with primary and borderline (meanwhile evolved technical term for “probably”) resectable cancer of the pancreatic head comparing primary surgery with neoadjuvant treatment followed by surgery, starting with randomization in 2003. Here, we report the full results of this trial, which was not picked up by the majority of the research community at the time the trial was conducted. As a consequence, the trial could not be completed and therefore shows a lack of statistical significance due to the poor recruiting rate. On the other hand, the reporting of negative trials (i.e., a trial with no clear interpretable results) is important to improve future trials.

An extended version of this manuscript including a detailed description of all methods employed in this study is provided as supplementary material.

## Patients and methods

### Study design and inclusion criteria

**Fig. 1:**
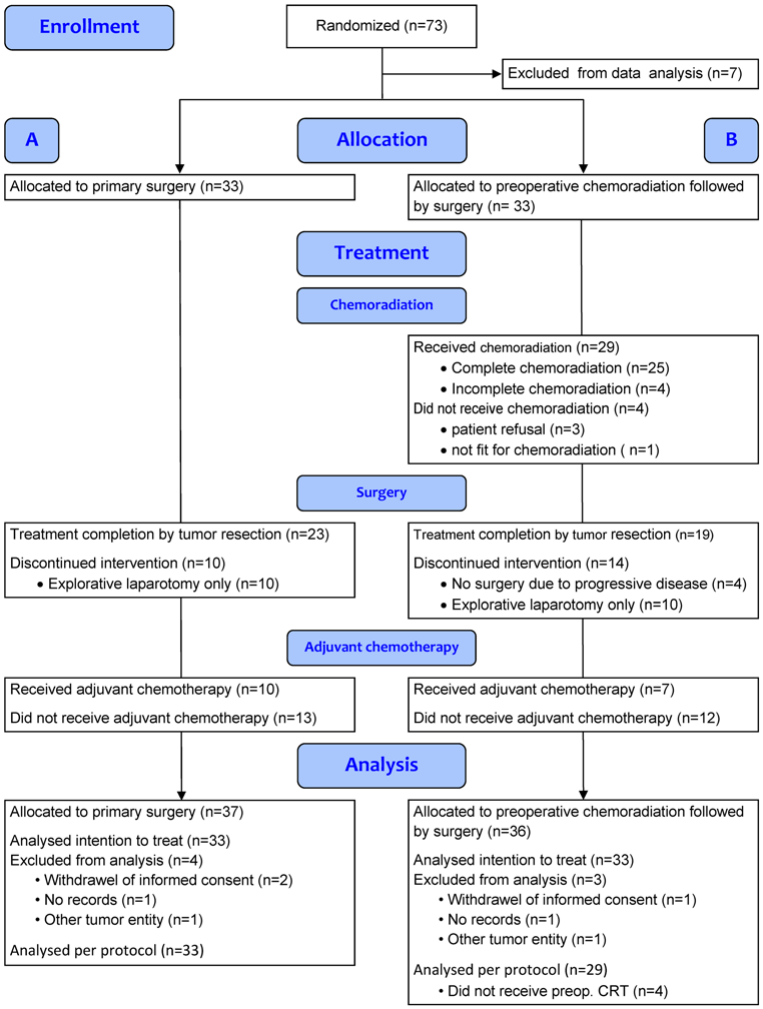
CONSORT diagram [36]

Patients with resectable, histology or cytology proven adenocarcinoma of the pancreatic head were randomized between surgery alone (Arm A) and neoadjuvant chemoradiation followed by surgery (Arm B; Fig. 1) [19].

Resectability was defined as no organ infiltration except the duodenum and maximal involvement of peripancreatic vessels ≤ 180° confirmed by high resolution CT [20]. All inclusion criteria are completely enlisted in Table S1.

The protocol was reviewed and funded by Deutsche Krebshilfe, approved “Gütesiegel A” by Deutsche Krebsgesellschaft and approved by the ethics committees of the participating institutions. All patients provided written informed consent.

### Treatment

#### Chemoradiation

Chemoradiation and surgery were described in detail in the trial protocol. Briefly patients in Arm B received 300 mg/m^2^ gemcitabine and 30 mg/m^2^ cisplatin on days 1, 8, 22, and 29 of radiotherapy. Three-dimensional treatment planning was mandatory for radiotherapy at 1.8 Gy to 55.8 Gy (tumor) or 50.4 Gy [regional lymph nodes, planning target volume (PTV ≤ 800 ml)] [21]. Dosis modifications in case of toxicity of chemotherapy were specified separately for gemcitabine and cisplatin. Criteria for patient withdrawal were also defined. Six weeks after chemoradiation, a restaging CT scan was scheduled.

#### Surgery

The surgical procedure was divided into the three steps: exploration, tumor resection, and lymph node dissection. At exploration, distant metastases had to be ruled out. Local resectability was assessed and in case of vascular tumor infiltration the decision to resect the tumor with adjacent vessels was completely left to the surgeon and the individual situation.

#### Adjuvant chemotherapy

In both arms, adjuvant chemotherapy according to the CONKO-001 study protocol was recommended in an amendment from 2005 [9].

### Assessment and follow-up

Resection specimens were graded and classified according to the sixth UICC TNM system [22]. Assessment of response to neoadjuvant therapy was based on contrast-enhanced restaging CT scans 6 weeks after completion of chemoradiation. RECIST criteria were used to classify response [23].

Acute toxicity and adverse effects were reported using the NCI common toxicity criteria v2.0 and RTOG/EORTC recommendations for classifying late toxic effects of radiotherapy [24, 25]. Perioperative complications were graded by Dindo’s classification [26].

Patients were followed up for at least 36 months at 3-month intervals until 2 years and 6-month intervals thereafter.

### End points, sample size, and statistical analysis

The primary endpoint of this trial was overall survival. In 2001, the study was planned in detail and the design was made to detect a change in mOS from 9.15 months in Arm A to 13.48 months in Arm B. A power of 80 % at the two-sided significance level of 5 % was chosen. It was estimated that 127 patients per arm would be required.

The statistical analysis was performed on all randomly assigned patients with pancreatic carcinoma and sufficient data. An intention-to-treat analysis calculated overall survival for all patients from random assignment. The Kaplan–Meier technique was used defining death by any cause as an event for estimating observed survival and the two-sided log-rank test to measure levels of significance. Time to progression was defined as time to first diagnosis of progression or recurrence or death of any cause and was analyzed for all patients. Comparisons between frequencies were performed using the "chi-square"  oder "χ²"^2^ test or, when appropriate, the Fisher’s exact test. *P*-values < 0.05 were considered significant.

## Results

### Patients

Between June 2003 and December 2009, 73 patients were recruited in eight university hospitals and tertiary referral centers in Germany and Switzerland. In December 2009, enrollment was terminated because of the poor recruitment rate. Seven patients (4 Arm A; 3 Arm B) were deemed ineligible because of withdrawal of consent, lack of data, and other tumor entity (Fig. 1). Two patients had metastases at randomization (*n* = 1 distant lymph nodes, *n* = 1 liver), both in Arm B. These patients were not excluded, as it reflects real life, where reviewing of initial data at the time of documentation in the case report form sometimes changes first impressions. Due to this low number of patients, the power for the formal statistical analysis was limited. All eligible patients were evaluable for survival. Patients’ characteristics are listed in Table 1.

**Table 1:** Patients’ demographic and baseline characteristics

Characteristics	Variable	Total *n* = 66 (%)	Surgery alone *n* = 33 (%)	CRT and surgery *n* = 33 (%)	*P* value
Patient variables					
Age (years)	Median (range)	63.9 (33–76)	65.1 (46–73)	62.5 (33–76)	0.62
Gender	Male	35 (53)	17 (52)	18 (55)	0.81
	Female	31 (47)	16 (48)	15 (45)	
KPS	100	13 (20)	7 (21)	6 (18)	0.36
	90	36 (54)	15 (46)	21 (64)	
	80	12 (18)	7 (21)	5 (15)	
	70	5 (8)	4 (12)	1 (3)	
Clinical tumor staging					
Clinical T category^a^	cT1	2 (3)	1 (3)	1 (3)	0.79
	cT2	30 (45)	15 (45)	15 (45)	
	cT3	33 (50)	17 (52)	16 (49)	
	cT4	1 (2)	0 (0)	1 (3)	
Clinical N category^a^	cN0	52 (79)	30 (91)	22 (67)	0.03
	cN1	14 (21)	3 (9)	11 (33)	
Clinical M category^a^	cM0	64 (97)	33 (100)	31 (94)	0.49
	cM1	2 (3)	0 (0)	2 (6)	
Clinical UICC stage^a^	I	29 (44)	16 (48)	13 (39)	0.31
	II	35 (53)	17 (52)	18 (55)	
	III	0 (0)	0 (0)	0 (0)	
	IV	2 (3)	0 (0)	2 (6)	
Procedures before randomization					
Explorative surgery before randomization	Exploratory surgery	36 (55)	17 (52)	19 (58)	0.62
	Laparoscopy	28 (42)	15 (46)	13 (39)	
	Laparotomy	8 (12)	2 (6)	6 (18)	
	Not done	30 (45)	16 (48)	14 (42)	
Biliary stent before randomization	Yes	57 (86)	28 (85)	29 (88)	1.0
	No	9 (14)	5 (15)	4 (12)	

*CRT* chemoradiation; *KPS* Karnofsky performance status

^a^According to UICC 2002

### Treatment

In Arm B, 29 of 33 patients received chemoradiotherapy. A total of 3 patients refused and 1 patient was not fit for chemoradiation, but all 4 patients underwent surgery. All 29 patients who underwent chemoradiation completed radiotherapy and were treated with a median of 55.8 Gy (range 45.0–57.6 Gy). Three patients had changes in chemotherapy on day 29 due to leukopenia. One patient received 5-fluorouracil/cisplatin instead of gemcitabine/cisplatin (local investigator judgment). All other patients received chemotherapy as planned. Toxicity of chemoradiation (Arm B) is shown in Table 2. During chemoradiotherapy and until surgery 15 severe adverse events were reported, mostly cholangitis requiring a change of stent (*n* = 9). Radiological response on restaging CT scan was rarely seen (*n* = 4 partial response), whereas most patients had no change (*n* = 8) or progression (*n* = 12; missing data *n* = 5).

**Table 2:** Acute toxicity^a^ of chemoradiotherapy

Parameter	*N*	Grade		
		0–2	3 (%)	4 (%)
Leukopenia	29	20	7 (24)	2 (7)
Thrombopenia	29	18	10 (35)	1 (3)
Anemia	29	27	1 (3)	1 (3)
Nausea/vomiting	28	18	10 (36)	–
Gastrointestinal bleeding	28	28^b^	–	–
Diarrhea	29	28	1 (3)	–
Elevated transaminases	29	23	5 (17)^c^	1 (3)
Elevated bilirubin	28	26	1 (4)	1 (4)^d^
Elevated alkaline phosphatase	29	24	5 (17)	–
Infection	29	24^e^	5 (17)^f^	–

^a^Toxicity was defined according to the National Cancer Institute Common Toxicity Criteria v2.0 [34]^b^1 of 28 patients grade 2

^c^4 of 5 patients due to cholangitis

^d^Due to cholangitis

^e^Grade1 and 2: *n* = 7 (5 patients cholangitis, 1 patient noro virus, 1 patient localization not known)

^f^4/5 cholangitis, 1 of 5 patients urinary tract infection

In the intention-to-treat analysis, in Arm A, 23 of 33 patients had tumor resection and 5 patients had vascular resections to achieve clinical R0 resection. Ten of 33 patients had an explorative laparotomy. In Arm B, 19/33 patients had tumor resection and 4 patients had extended surgery to achieve R0 resection. Ten of 33 patients had an explorative laparotomy. Four patients had no surgery due to progressive disease. Resection rates between the arms were not different (*P* = 0.31). In Arm B, 3 of 4 patients without chemoradiation had tumor resection; 1 patient had liver metastases at exploration.

Of importance, patients in Arm B did not have elevated rates of high-grade post-operative complications (Table 3).

**Table 3:** Postoperative complications

		Dindo’s grade [36]				
		All (1–5)	1–2	3a/3b	4a/4b	5
Surgery alone (Arm A; *n* = 33)		32	17	9	4	2
As treated (*n* = 37)	Resection (*n* = 26)	23	12	6	4	1
	Exploration (*n* = 11)	9	5	3	0	1
CRT and surgery (Arm B; *n* = 33)		22	16	6	0	0
As treated (*n* = 29)	Resection (*n* = 16)	14	9	5	0	0
	Exploration (*n* = 9)	8	7	1	0	0
	(no surgery *n* = 4)	–	–	–	–	–
Total (*n* = 66)		54	33	15	4	2
	Resection (*n* = 42)	37	21	11	4	1
	Exploration (*n* = 20)	17	12	4	0	1
	No surgery (*n* = 4)		–	–	–	–

*CRT* chemoradiotherapy

One patient died as the result of an intraoperative myocardial infarction after tumor resection (Arm A) and 1 patient died due to sepsis possibly due to cholangitis after explorative laparotomy (Arm A). One patient had insufficiency of the pancreaticojejunal anastomosis followed by multiple organ dysfunction (grade 4b, Arm A; none in Arm B). The most severe post-operative complications after chemoradiation were grade 3b (intervention under general anesthesia) due to intraabdominal abscess/fluid retention (*n* = 4) or insufficiency of the choledochojejunal anastomosis (*n* = 1).

In Arm A, 10 of 23 patients had adjuvant chemotherapy and in Arm B 7 of 19 patients.

### Outcome

**Fig. 2:**
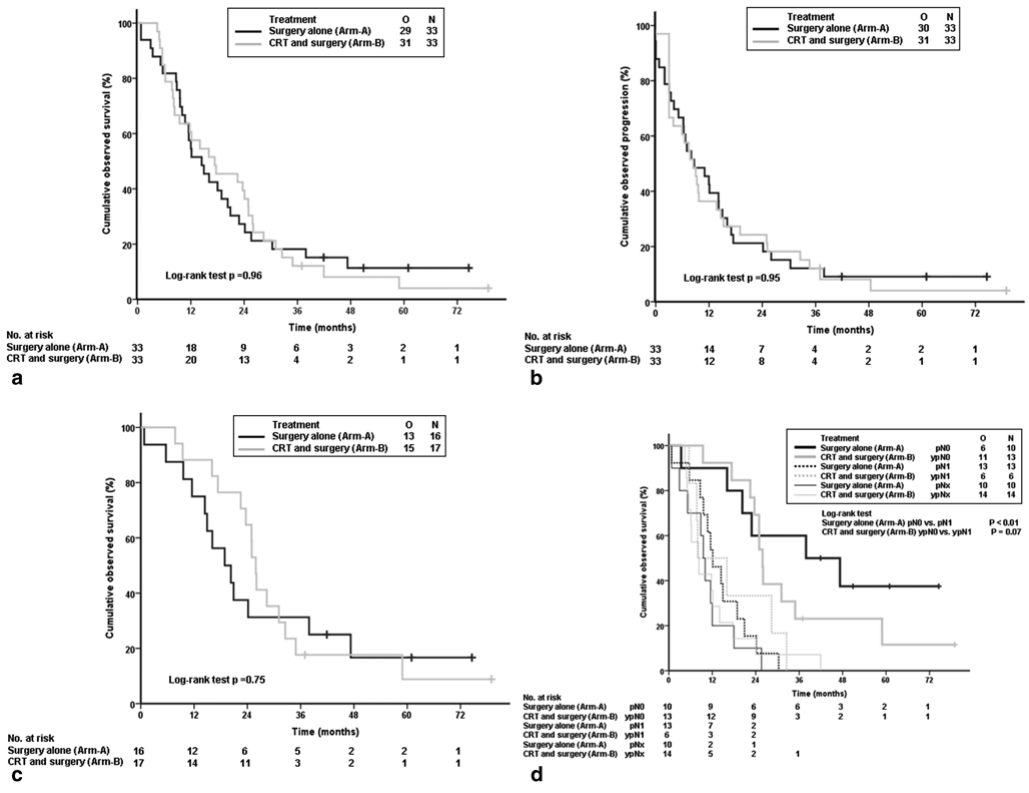
Kaplan–Meier curves (intention to treat analysis) for **a** overall survival, **b** time to progression, **c** overall survival after R0 resection, and **d** overall survival according to (y)pN status. *CRT* chemoradiation; *O* events [**a**, **c** **,** and **d** deaths or **b** progression of disease] observed; *N* overall number; *pNx* no tumor resection (**d**)

The median follow-up for all living patients was 61 months (range 37–79 months). There were 29 deaths in Arm A and 31 deaths in Arm B. At intention-to-treat analysis mOS between the two arms was not significantly different for all patients irrespective of resection status (Arm A, 14.4 months; Arm B 17.4 months; *P* = 0.96; Fig. 2a).

After resection, mOS was 18.9 months (Arm A) versus 25.0 months (Arm B; *P* = 0.79; intention-to-treat analysis). Time to progression measured 8.7 versus 8.4 months (Arm A versus Arm B; *P* = 0.95; Fig. 2b).

Pathohistological diagnosis of pancreatic adenocarcinoma at biopsy was confirmed in 42 of 44 resection specimens. One distal choledochal adenocarcinoma (Arm B) and 1 duodenal adenocarcinoma (Arm A) were excluded from all analyses. R0 resections were achieved in 16 of 33 patients versus 17 of 33 patients (Arm A versus Arm B; *P* = 0.81), and mOS was 18.9 months (Arm A) versus 25.9 months (Arm B; *P* = 0.75; Fig. 2c). Nodal status was (y)pN0 in 10 of 33 patients and 13 of 33 patients in Arm A and Arm B, respectively (*P* = 0.44). (y)pN0-status resulted in significantly longer mOS in Arm A (Fig. 2d). Four patients had pathologically proven distant metastases resected [Arm A *n* = 2 (lymph node, duodenum); Arm B *n* = 2 (lymph node)]. Pathological results for resected patients are listed in Table 4.

**Table 4:** Pathological staging

Characteristic	Variable	Surgery alone (Arm A)	CRT and surgery (Arm B)
		*N* = 23	*N* = 19
Pathological T category^a^	(y)pT1	0	2
	(y)pT2	2	2
	(y)pT3	20	15
	(y)pT4	1	0
Pathological N category^a^	(y)pN0	10	13
	(y)pN1	13	6
Pathological M category^a^	(y)pM0	21	17
	(y)pM1	2	2
Pathological UICC stage^a^	(y)pI	1	4
	(y)pII	19	13
	(y)pIII	1	0
	(y)pIV	2	2
Grading	G1	0	0
	G2	11	9
	G3	10	8
	G4	2	1
	Not specified	0	1
Resection margin	R0	16	17
	R1	7	2

*CRT* chemoradiotherapy

^a^According to UICC 2002

## Discussion

The planning of this trial was started in 1999 with activation in 2003 before neoadjuvant treatment had become standard for other diseases (e.g., rectal carcinoma) and therefore had to overcome resistance by physicians and patients likewise against the idea of neoadjuvant treatment as such. Additionally, competing adjuvant trials (CONKO-001 [9], ESPAC-3 [11]) resulted in lower participation. Another issue was histological or cytological proof of disease before randomization. To overcome this obstacle to recruitment, the protocol allowed randomization after histological proof during explorative laparotomy. However, to our knowledge this remains the first planned and evaluated multicenter RCT comparing immediate surgery with surgery after neoadjuvant therapy in resectable pancreatic cancer, defined as vascular abutment of less than 180°. But due to low patient numbers this is a negative trial and no clear conclusion can be drawn from underpowered data and whether there is an advantage for one therapy strategy or not.

The following issues of a randomized controlled trial for resectable pancreatic cancer have to be addressed in future trial protocols: working in interdisciplinary teams, predicting resectability, definition of vascular resection aims, definition of criteria for cancelling tumor resection during explorative laparotomy, and adjuvant chemotherapy. One of the main problems remains how to predict resectable tumor stage at diagnosis as 20 % of tumors without contact to the peripancreatic vessels at diagnosis were not resected with and without neoadjuvant chemoradiation (data not shown). Clearly, the new definition of borderline resectable pancreatic cancer is helpful, but has to be evaluated in future trials. A further point of discussion is the different judgment between centers with reference to cancelling surgery, as only one center abandoned resection of the tumor after detection of distant lymph node metastasis (2 patients) or did not proceed to surgery when progression (locally, distant, clinically) at restaging after chemoradiation was seen (data not shown).

The initially mandatory laparoscopy was reclassified as optional due to objections of potential trial participants in a 2004amendment. Altogether, surgical staging was conducted only in 54 % of all patients and should be considered in further trials on preoperative treatment strategies [15].

The closest possible comparison of this trial is with adjuvant treatment, especially with the CONKO-001 trial conducted in the same population and with an observation arm [9, 27]. However, the fundamental difference between the reported trial here and adjuvant treatment is that the latter only includes patients after resection and pathological staging, whereas in this study 24 of 68 patients (35 %) had reasons preventing curative resection despite the suggested resectability at staging. Median overall survival in the CONKO-001 trial was 20.2 and 22.1 months (control versus adjuvant gemcitabine, *P* = 0.06). This compares well with the mOS of patients with resections in this trial (18 and 25 months; Arm A versus Arm B). In CONKO-001, resection margin status was a negative prognostic marker in the observation arm (mOS 20.8 and 14.1 months R0 versus R1). Recent reports about the lack of prognostic significance of margins might be related to frequent underreporting of R1 status because series with high R1 resection rates correlated with the highest prognostic value of margin status. Therefore, higher R0 resection rates after neoadjuvant treatment are expected to have an impact on survival [17, 18, 28–30].

Neoadjuvant treatment did not show an effect in this strongly underpowered trial due to underrecruitment, but on the other hand was a suitable instrument for selecting patients for surgery. Patients with initially unknown distant metastases might be unmasked by preoperative therapy and hence spared from surgery [16]. In this trial, all patients with neoadjuvant treatment survived at least 3 months, whereas after primary surgery 3 of 34 patients died within this timeframe. Additionally, less severe complications were seen after chemoradiation therapy, probably due to induction of fibrosis, which improves the suitability of pancreatic tissue for anastomosis. A recent meta-analysis also found similar perioperative morbidity with and without neoadjuvant treatment [18].

Toxicity of chemoradiotherapy was well manageable in this trial. The well-known risk of biliary stent dysfunction was managed by prompt stent replacement, but was the most frequent reason for severe adverse events. Hematologic toxicity of gemcitabine-based CRT is directly related to radiotherapy volume and, therefore, volumes were strictly limited [31–33]. Additionally, consequent supportive therapy may explain the improved tolerability of treatment in this trial compared to others avoiding loss of weight which was described to be a negative prognostic factor after neoadjuvant chemoradiotherapy [34]. The patients in this trial were treated with 3D-conformal plans which have recently been shown to be equally effective and not significantly more toxic as IMRT plans in the neoadjuvant setting [35].

Furthermore, predicting resectability based on CT scans was difficult. Thus, the CONKO-007 (NCT01827553) trial will study the role of chemoradiation in borderline resectable and nonresectable pancreatic cancer. A panel of highly experienced surgeons will review all CT scans before registering to the trial and at restaging and give their statement about resectability. With the experience of such a trial, the criteria of R0 resectability will be evaluated and adjusted. Then after knowing the significance of chemoradiation for locally advanced and borderline resectable pancreatic cancer, the next step might be a phase II trial testing the R0 resectability with neoadjuvant therapy.

## Conclusion

Presented in this article are the results of a RCT implicating the strategy of multimodal therapy for (borderline) resectable pancreatic cancer which was visionary at the time of planning and conduction of the trial; it was nearly 15 years ahead of its time before this approach was again implemented into prospective trials in Europe. In the meantime, the conditions for conducting interdisciplinary trials have improved much due to governmental regulations and nationwide implementation of certified cancer centers with interdisciplinary tumor boards. The improvement of interdisciplinary study structures and the lack of better therapies evolving in the meantime led to boycotting this trial to copying the treatment strategy of neoadjuvant chemoradiation with starting a nearly identical study protocol in August 2013 (NCT01900327). Prediction of resectability preoperatively is still an unresolved problem and the long-term results of treatment for pancreatic cancer are still frustrating even after complete tumor resection. Thus, at the moment we do not have a better choice but to investigate new treatment strategies suitable for as many patients with pancreatic cancer as possible.

### Acknowledgments

We are grateful to the patients who participated in this study. We thank all of the investigators who participated in this study: N. Christen, S. Kißenkötter, H. Lauer, V. Lück, Krankenhaus Dresden-Friedrichstadt, Dresden; B. Adamietz, A. Agaimy, U. Baum, R. Croner, A. Dimmler, M. Geiger, S. Herold, R. Janka, A. Kergaßner, P. Klein, S. Krüger, M. Lindenberg, W. Melzner, T. Papadopoulos, J. Pelz, A. Schlabrakowski, U. von Linden, C. Weiß, M. Zeilinger, Universitätsklinikum Erlangen, Erlangen; C. Gog, D. Imhoff, C. Wullstein, Universitätsklinikum Frankfurt, Frankfurt/Main; H. Bockhorn, A. Hildebrand, Krankenhaus Nordwest, Frankfurt/Main; J. Behrbohm, K. Gumpp, J. Hauss, A. Liebmann, Universitätsklinikum Leipzig, Leipzig; H. J. Schlitt, C. Zühlke, Universitätsklinikum Regensburg, Regensburg; T. Horbach, Krankenhaus Schwabach, Schwabach; S. Bischofberger, P. Folie, D. Hausmann, D. Köberle, J. Lange, C. Meyenberger, I. Neuweiler, G. Ries, M. Zünd, Kantonsspital St. Gallen, St. Gallen.

### Funding

This work was supported by Deutsche Krebshilfe (70–3046-Ho 2 to W.H.) and Verein zur Förderung des Tumorzentrums der Universität Erlangen-Nürnberg e. V.

## Electronic supplementary material

(PDF 604 kb)
