# Supplementary material for: Neoadjuvant chemoradiation therapy with gemcitabine/cisplatin and surgery versus immediate surgery in resectable pancreatic cancer: Results of the first prospective randomized phase II trial
Source: Strahlenther Onkol. 2014 Sep 25;191(1):7–16. doi: 10.1007/s00066-014-0737-7 (PMC4289008; doi:10.1007/s00066-014-0737-7)
Supplement: Supplementary file 1 — (PDF 604 kb) [file 66_2014_737_MOESM1_ESM.pdf]

Strahlenther Onkol 2014
Supplementary Data
DOI 10.1007/s00066-014-0737-7
Received: 11 January 2014
Accepted: 23 July 2014
© Springer-Verlag Berlin Heidelberg 2014

H. Golcher*[1] · T.B. Brunner*[2, 3] · H. Witzigmann[4, 5] · L. Marti[6] · W.O. Bechstein[7] · C. Bruns[8,9] · H. Jungnickel[5] · S. Schreiber[4] · G.G. Grabenbauer[2, 10] · T. Meyer[1, 11] · S. Merkel[1] · R. Fietkau[2] · W. Hohenberger[1]

***The first and second authors contributed equally to the manuscript.**

[1]Department of Surgery, University Hospital Erlangen, Erlangen, Germany
[2]Department of Radiation Oncology, University Hospital Erlangen, Erlangen, Germany
[3]Department of Radiation Oncology, University Hospital Freiburg, Freiburg, Germany
[4]Department of Surgery, University Hospital Leipzig, Leipzig, Germany
[5]General Surgery, Hospital Dresden-Friedrichstadt, Dresden, Germany
[6]General Surgery, Hospital of Kanton St. Gallen, St. Gallen, Switzerland
[7]Department of Surgery, University Hospital Frankfurt, Frankfurt/Main, Germany
[8]Department of Surgery – Hospital Campus Großhadern, University Hospital Munich, Munich, Germany
[9]Department of Surgery, University Hospital Magdeburg, Magdeburg, Germany
[10]Department of Radiation Oncology, Hospital Coburg, Coburg, Germany
[11]General Surgery, Hospital Ansbach, Ansbach, Germany

This is the extended version of the manuscript "Golcher H, Brunner TB, et al (2014) Neoadjuvant chemoradiation therapy with gemcitabine/ cisplatin and surgery versus immediate surgery in resectable pancreatic cancer. Results of the first prospective randomized phase II trial. (ISRCTN78805636). Strahlenther Onkol" including a detailed description of all methods employed in this study, reporting the complete results and featuring an introduction/ discussion at full length provided as supplementary material to the original paper.

Neoadjuvant chemoradiation therapy with gemcitabine/ cisplatin and surgery versus immediate surgery in resectable pancreatic cancer. Results of the first prospective randomized phase II trial. (ISRCTN78805636).

Background

Survival rates of patients with pancreatic cancer have improved only marginally during the last 30 years with a 5-year survival rate of only 6%.[1] In contrast the prognosis of patients with rectal carcinoma has improved substantially during the same timeframe.[2] This progress was caused by standardizing surgical therapy[3] worldwide and by the implementation of multimodal therapy.[4, 5, 6] Moreover in rectal cancer it was found early, that a clear circumferential margin is important and that even margins below 1mm cause a significant increase in the rate of local recurrence.[7] All these measures caused a decline in local recurrence from 50% to about 10% and an increase of 5-year survival rates up to more than 50% worldwide. This progress led to the hypothesis that in analogy the much worse prognosis of ductal adenocarcinoma of the pancreas might be improved.

In pancreatic cancer only in recent years it became obvious, that up to 75% of macroscopically clear resection margins during more precisely work up became R1 resections with tumor extensions up to the circumferential margin[8], which classifies the result of the surgery as palliative.[9, 10] These patients have no chance of cure but have surgery with the significant risk of postoperative complications of up to 40% and a postoperative letality of up to 5%.[11, 12, 13]

Adjuvant therapy has been tested in a series of RCT (randomized controlled trial) phase III trials, the most important of these are the ESPAC-1 trial, the CONKO-001 trial, the RTOG 97-04 trial and the ESPAC-3 trial.[14, 15, 16, 17] But these trials were still running or results were not yet available when the here described trial was planned and conducted. The results of these trials led to a change in standard treatment recommending adjuvant treatment with chemo-therapy since 2007 in Germany.[18]

The concept of neoadjuvant rather than adjuvant treatment in pancreatic cancer appears attractive for a number of reasons. Firstly, up to 30% of the tumors staged as resectable cannot be resected due to undetected metastatic disease or underestimated

tumor contact to peripancreatic vessels detected not until explorative laparotomy.[19] Secondly, up to 30% of the patients cannot receive adjuvant therapy because of poor post-operative performance status.[20] Both groups of patients are not included into adjuvant trials, though improving overall survival in both arms (adjuvant therapy vs. no adjuvant therapy) by simple patient selection. Neoadjuvant treatment is thought to be better tolerated, than adjuvant treatment and avoids postsurgical morbidity in patients with rapidly metastasizing tumors. Non-randomized trials using the neoadjuvant approach support this rationale: Median OS beyond 30 months for patients after neoadjuvant treatment and tumor resection were described in a number of retrospective data analyses.[21, 22, 23, 24]

Therefore, in 1999 we started to plan this multi-center randomized phase II-study in patients with locally resectable cancer or probably locally resectable cancer of the pancreatic head with strict imaging eligibility criteria defining vascular involvement. Meanwhile the technical term “borderline resectable cancer” evolved for “probably resectable cancer”. High-quality chemoradiation aimed to maximize tolerance and efficacy of neoadjuvant treatment, both of which have been problematic in previous trials of chemoradiation in pancreatic cancer.

To our knowledge, this is the first RCT for patients with primary and

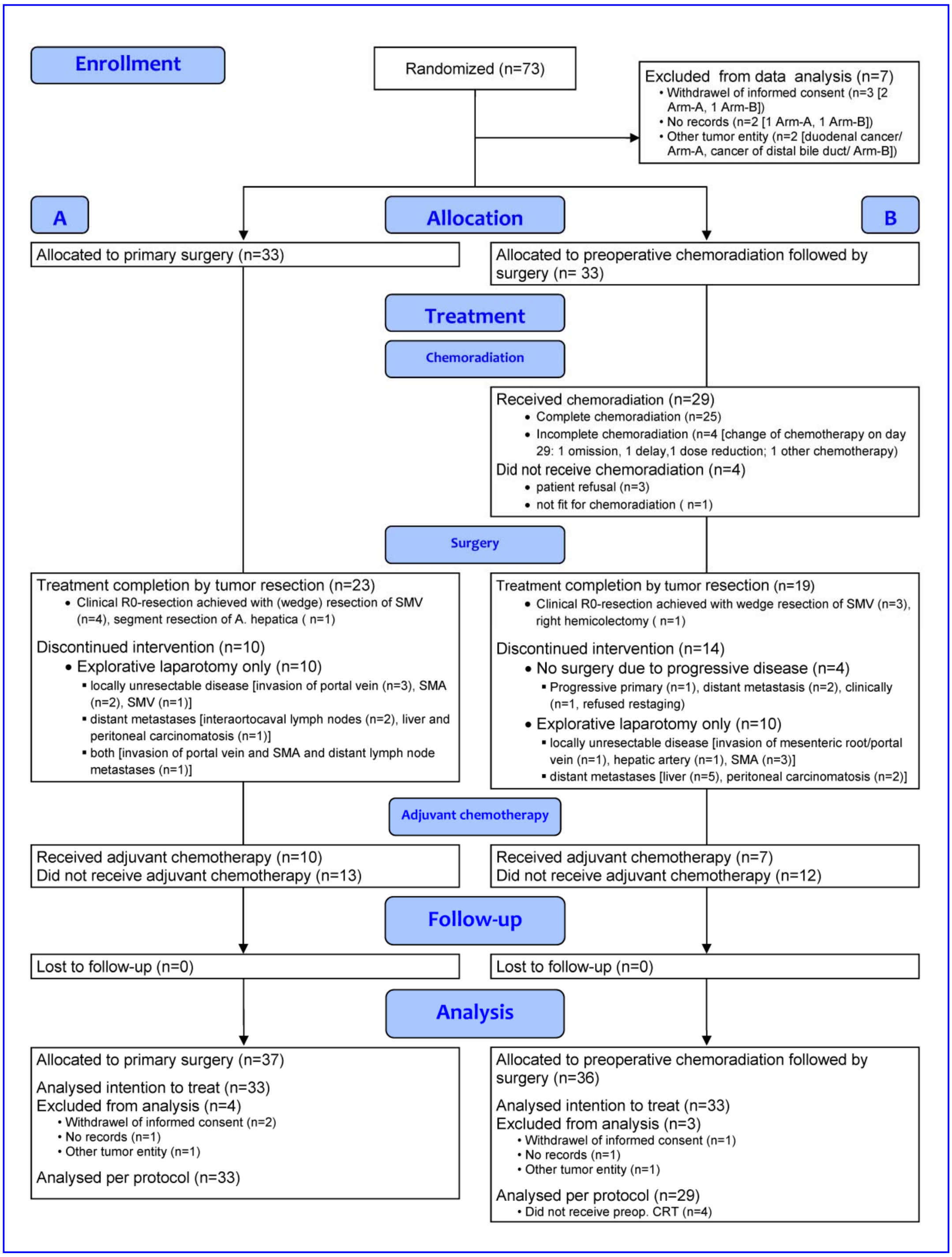

Fig.1 ◀ CONSORT Diagram[47]

borderline resectable cancer of the pancreatic head comparing primary surgery with neoadjuvant treatment followed by surgery, starting with randomization in 2003. Here, we report the full results of this trial, which was not picked up by the majority of the research community at the time of the conduction of the trial. In consequence, the trial could not be completed and therefore shows a lack of statistical significance due to poor accrual rate. On the other hand the reporting of negative trials (i.e. a trial with no clear interpretable results) is important for the improvement of the conduction of new trials.

Patients and methods

Study design and inclusion criteria

Patients with resectable, histology or cytology proven adenocarcinoma of the pancreatic head were randomized between surgery alone (Arm-A) and neoadjuvant chemoradiation followed by surgery (Arm-B) (Fig. 1).[25] Randomization was carried out centrally by fax by an independent contract research organization with stratification according to the clinical center and according to whether or not a laparoscopy has been performed (amendment 2004). Randomization was performed in blocks with randomly selected sizes of blocks of 4 and 6 patients.

Resectability was defined as no organ infiltration except the duodenum and maximal involvement of peripancreatic vessels ≤180° (portal vein, confluent, superior mesenteric artery (SMA), celiac trunk with its major branches splenic artery and hepatic artery, superior mesenteric vein (SMV)) confirmed by high resolution CT analogue to Lu et al.[26] Surgical staging by laparoscopy or laparotomy to exclude distant metastases prior to randomization was at the discretion of the local investigator after an amendment in 2004. Other recommended tests before inclusion into the study were physical examination, hematology, biochemistry, CA19-9 and chest X-ray. All inclusion criteria are completely enlisted in Tab. S1.

The protocol was reviewed and funded by Deutsche Krebshilfe, approved "Gütesiegel A" by Deutsche Krebsgesellschaft and approved by the ethics committees of the participating institutions. All patients provided written informed consent.

Tab. S1 Inclusion/ exclusion criteria

Inclusion criteria	Exclusion criteria
• Histologically confirmed ductal adenocarcinoma of the pancreatic head (tumors of the pancreatic head on the right to the left edge of the superior mesenteric vein including the uncinate process), stages I-IV A according to UICC 1997. If confirmation is not possible by endoscopic biopsy or by endoscopic cytology (brush cytology with tumor cell nests), CT or ultrasound-guided biopsy should be carried out (up to 3 attempts).<br>• No infiltration of extrapancreatic organs with the exception of the duodenum<br>• Pancreatic tumor confirmed by high-resolution spiral CT (layer thickness preferably 3 mm) that is classified as resectable or probably resectable by an experienced pancreatic surgeon for the following reasons: vascular involvement ≤ 180° of one of the peripancreatic major vessels (portal vein, confluent of superior mesenteric vein and splenic vein, superior mesenteric artery, celiac trunk with its major branches splenic artery and hepatic artery, superior mesenteric vein) (criteria according to Lu et al., 1997).<br>• No more than 1 enlarged (> 1cm) regional lymph node in thin slice spiral CT, without signs of vessel infiltration (omitted in amendment 2005)<br>• No distant metastasis<br>• No peritoneal spread<br>• Age at treatment initiation at least 18 years and not older than 75 (upper age limit omitted in amendment 2005)<br>• Karnofsky index ≥ 70<br>• Written informed consent of the patient	• Ampullary carcinoma (tumors originating from the ampulla, the papilla or at the junction of the ampulla and the papilla)<br>• Carcinoma of the pancreatic corpus or tail (tumors between the left edge of the superior mesenteric vein and the left edge of the aorta or between the left edge of the aorta and the splenic hilum)<br>• Non-ductal adenocarcinoma of the pancreas (e.g. cystadenocarcinoma, neuroendocrine tumors, etc.)<br>• Tumor-specific prior treatment<br>• Recurrent tumor<br>• Peritoneal spread<br>• Distant metastases<br>• 2 or more enlarged lymph nodes (> 1cm) with suspicion of metastatic spread based on morphology in CT scan (omitted in amendment 2005)<br>• Infiltration of extrapancreatic organs with the exception of the duodenum<br>• Vascular involvement > 180° of at least one of the major peripancreatic vessels (portal vein, confluent of the superior mesenteric vein and the splenic artery, superior mesenteric artery, the coeliac trunk, the superior mesenteric vein), stenosis or occlusion of the above mentioned vessels. Precondition of resectability only if vascular resection is performed (including portal vein and superior mesenteric vein).<br>• Prior or synchronous malignancy (except: non-melanomatous skin cancer and curatively treated carcinoma in situ of the uterine cervix and tumor treated by surgery alone with 10 years of disease-free survival)<br>• Participation in a clinical trial within the last three months prior to inclusion<br>• Liver cirrhosis with thrombocytes < 100,000 / mm^3 or PTT < 70%<br>• Serum creatinine > 1.5 mg/dl, creatinine clearance < 70 ml/min (24 hour collection phase)<br>• Severe cardio-pulmonary concomitant disease (cardiac insufficiency NYHA III/IV, arrhythmia Lown III/IV, pathological findings at ultrasound (pathological ejection fraction), respiratory global insufficiency) or any other serious disease, that could interfere with complete therapy as rated by the surgeons or radiation oncologists who participate in the treatment (such as severe cholangitis or jaundice that cannot be controlled despite implanting a stent – example added in amendment 2004).<br>• HIV infection<br>• Pregnancy ordesire for children in female patients<br>• Age under 18 years or over 75 (upper age limit omitted in amendment 2005)<br>• Karnofsky performance status < 70<br>• Justified doubt as to the understanding or contractual capacity of the patient

Treatment

Chemoradiation

Chemoradiation and surgery were described in detail in the trial protocol. Briefly patients in Arm-B received 300 mg/m2 gemcitabine and 30 mg/m2 cisplatin on days 1, 8, 22 and 29 of radiotherapy. The combination of gemcitabine and cisplatin was chosen, due to good experience with this regimen combined with radiotherapy at the time of study planning in 2000 and beyond.[27, 28] The sideeffects of both substances do only slightly overlap. 3D-treatment planning was mandatory for radiotherapy at 1.8 Gy to 55.8 Gy (tumor) or 50.4 Gy (regional lymph nodes, PTV ≤800 mL).[29] Supportive therapy consisted of dietary advice, anemia compensation <11 g/dL hemoglobin during chemoradiation, adequate analgesia and anti-emetics as well as

Tab. 1 Patient`s demographic and baseline characteristics					
**Characteristics**	**Variable**	**Total** *n*=66 (%)	**Surgery alone** *n*=33 (%)	**CRT and surgery** *n*=33 (%)	**P value**
**Patient variables**					
Age (years)	Median (Range)	63.9 (33-76)	65.1 (46-73)	62.5 (33-76)	0.62
Gender	Male	35 (53)	17 (52)	18 (55)	0.81
	Female	31 (47)	16 (48)	15 (45)	
KPS	100	13 (20)	7 (21)	6 (18)	0.36
	90	36 (54)	15 (46)	21 (64)	
	80	12 (18)	7 (21)	5 (15)	
	70	5 (8)	4 (12)	1 (3)	
**Clinical tumor staging**					
Clinical T category*	cT1	2 (3)	1 (3)	1 (3)	0.79
	cT2	30 (45)	15 (45)	15 (45)	
	cT3	33 (50)	17 (52)	16 (49)	
	cT4	1 (2)	0 (0)	1 (3)	
Clinical N category*	cN0	52 (79)	30 (91)	22 (67)	0.03
	cN1	14 (21)	3 (9)	11 (33)	
Clinical M category*	cM0	64 (97)	33 (100)	31 (94)	0.49
	cM1	2 (3)	0 (0)	2 (6)	
Clinical UICC stage*	I	29 (44)	16 (48)	13 (39)	0.31
	II	35 (53)	17 (52)	18 (55)	
	III	0 (0)	0 (0)	0 (0)	
	IV	2 (3)	0 (0)	2 (6)	
**Procedures before Randomization**					
Explorative surgery before randomization	Expl. surgery	36 (55)	17 (52)	19 (58)	0.62
	Laparoscopy	*28 (42)*	*15 (46)*	*13 (39)*	
	Laparotomy	*8 (12)*	*2 (6)*	*6 (18)*	
	Not done	30 (45)	16 (48)	14 (42)	
Biliary stent before randomization	Yes	57 (86)	28 (85)	29 (88)	1.0
	No	9 (14)	5 (15)	4 (12)	

Abbreviations: CRT, chemoradiation; KPS, Karnofsky performance status
* According to UICC 2002

implantation of a bile duct stent if bilirubin exceeded 1,5 mg/dl. Dosis modifications in case of toxicity of chemotherapy (hematologic, renal, neurologic, other) were specified in the trial protocol separetaly for gemcitabin and cisplatin. Criteria for patient withdrawal (for example: febrile neutropenia with sepsis, Karnofsky-Index < 50%, interruption of radiotherapy for > 10d) were specified in the trial protocol, too. Six weeks after chemoradiation, a restaging CT-scan was scheduled.

Surgery

The surgical procedure was described in detail in the protocol, divided into the three steps of exploration, tumor resection and lymph node dissection. At exploration distant metastases had to be ruled out, fresh frozen biopsies were taken as appropriate. Local resectability was assessed by dissection of SMV, portal vein and common hepatic artery, occasionally preliminary dissection of SMA. In case of vascular tumor infiltration the decision to resect the tumor with adjacent vessels was completely left to the surgeon and the individual situation.
The minimal requirements for tumor resection were: Partial duodeno-pancreatectomy (± pylorus-preserving), transection of the pancreas minimally at the level of the left edge of the portal vein and transection of the common hepatic duct slightly distally to the junction of the right and left hepatic duct. The extent of lymph node dissection was, in short, at least: complete dissection of hepaticoduodenal ligament, common hepatic artery, circular dissection of the celiac trunk and right and dorsal of the SMA from its origin until the derivation of the first jejunal artery to the left. Preservation of the nervous plexus at the trunk of the SMA was mandatory. At least three lymph nodes had to be excised from interaortocaval lymph nodes (between the left renal vein and the inferior mesenteric artery) for staging purposes. All three resection planes of the specimen were recommended to be analyzed histologically at intraoperative fresh frozen section. It was mandatory to perform histological analysis of the postoperatively paraffin embedded tissue to exclude positive margins (R1-resection).

Adjuvant chemotherapy

In both arms, adjuvant chemotherapy according to the CONKO-001 study protocol was recommended in an amendment from 2005.[15]

Assessment and follow-up

Resection specimens were graded and classified according to the fifth and sixth UICC TNM system (1997 and 2002) with documentation of resection margins, tumor size, number of examined and involved lymph nodes, and presence of lymphatic, venous or perineural invasion.[30, 31] Tumor regression was classified for tumors and lymph nodes in Arm-B.[32]
Assessment of response to neoadjuvant therapy was based on contrast enhanced re-staging CT-scans six weeks after completion of chemoradiation. RECIST criteria were used to classify response.[33] Involvement of peripancreatic vessels was also reassessed.
Acute toxicity and adverse effects were reported using the National Cancer Institute Common Toxicity Criteria v2.0 and RTOG / EORTC

recommendations for classifying late toxic effects of radiotherapy.[34, 35] Perioperative complications and corresponding interventions were documented and graded by Dindo`s classification.[36]
Patients were followed up for at least 36 months at 3-month-intervals until 2 years and 6-month-intervals thereafter. Follow-up consisted of physical examination, hematology, biochemistry and CA19-9 as well as abdominal CT-scan and chest X-ray every 6 months.

End points, sample size and statistical analysis

The primary endpoint of this trial was overall survival. In 2001 the study was planned in detail and the design was made to detect a change in mOS from 9.15 months in Arm-A to 13.48 months in Arm-B. The survival rates were derived from the analysis of data from 1995 to 2000 at the Tumor Registry of the Department of Surgery, University Hospital Erlangen. A power of 80% at the two-sided significance level of 5% was chosen. It was estimated that 127 patients per arm would be required.
The statistical analysis was performed on all randomly assigned patients with pancreatic carcinoma and sufficient data. An intention-to-treat analysis calculated overall survival for all patients from random assignment. The Kaplan-Meier technique was used defining death by any cause as an event for estimating observed survival and the two-sided log-rank test to measure levels of significance. Disease free survival was defined as time from randomization until diagnosis of local recurrence, metastases or death of any cause (resected patients only). Time to progression was defined as time to first diagnosis of progression or recurrence or death of any cause and was analyzed for all patients. Comparisons between frequencies were performed using the chi-square test or, when appropriate, the Fisher’s exact test. P-values <0.05 were considered significant. Statistical Package for the Social Sciences version 21.0 was used to perform data analyses.

Results

Patients

Between June 2003 and December 2009, 73 patients were recruited in 8 university hospitals and tertiary referral centers in Germany and Switzerland. In December 2009, enrollment was terminated because of the poor recruitment rate. Seven patients (4 Arm-A; 3 Arm-B) were deemed ineligible because of withdrawal of consent, lack of data and other tumor entity (two patients). Both patients were intra-/ postoperatively diagnosed in spite of the histological proven diagnosis of adenocarcinoma of the pancreatic head before inclusion into the trial (Fig. 1). Two patients had metastases at randomization (n=1 distant lymph nodes, n=1 liver), both in Arm-B. These patients were not excluded, as it reflects real life, where reviewing of initial data at the time of documentation in the case report form sometimes changes first impressions. Due to this low number of patients, the power for the formal statistical analysis was limited. All eligible patients were evaluable for survival. Patient characteristics are listed in Tab. 1.

Treatment

In Arm-B, median start of treatment was 13 days after randomization (range 0-31days). 29/33 patients received chemoradiotherapy. Three patients refused and one was not fit for chemoradiation, but all four underwent surgery. All 29 patients who underwent chemoradiation completed radiotherapy and were treated with a median of 55.8 Gy (range 45.0-57.6). Chemoradiation took 36 – 49 days (median 44 days). Three patients had changes in chemotherapy on day 29 due to leukopenia. One patient received 5-fluorouracil/ cisplatin instead of gemcitabine/ cisplatin (local investigator judgement). All other patients received chemotherapy as planned, resulting in a dose intensity of 99% for cisplatin and 95% for gemcitabin. Toxicity of chemoradiation (Arm-B) is shown in Tab. 2. During chemoradiotherapy and until surgery 15 severe adverse events were reported, mostly cholangitis requiring a change of stent (n=9). Radiological response on re-staging CT-scan was rarely seen (n=4 partial response), whereas most patients had no change (n=8) or progression (n=12; missing data n=5). In Arm-A patients had surgery 4 days (median) after randomization (range 0-19 days). In the intention-to-treat analysis, in Arm-A, 23/33 had tumor resection and five patients had vascular resections to achieve clinical R0-resection. Ten of 33 patients had an explorative laparotomy. In Arm-B

Tab. 2 Acute toxicity[a] of chemoradiotherapy

		Grade		
**Parameter**	**n**	**0-2**	**3 (%)**	**4 (%)**
Leukopenia	29	20	7 (24)	2 (7)
Thrombopenia	29	18	10 (35)	1 (3)
Anemia	29	27	1 (3)	1 (3)
Nausea/Vomiting	28	18	10 (36)	-
Gastrointestinal bleeding	28	28[b]	-	-
Diarrhea	29	28	1 (3)	-
Elevated Transaminases	29	23	5 (17)[c]	1 (3)
Elevated Bilirubin	28	26	1 (4)	1 (4)[d]
Elevated Alkaline phosphatase	29	24	5 (17)	-
Infection	29	24[e]	5 (17)[f]	-

[a]Toxicity was defined according to the National Cancer Institute Common Toxicity Criteria v2.0.[34]
[b]1/28 patients grade 2; [c]4/5 patients due to cholangitis; [d]due to cholangitis; [e]grade 1 and 2: n=7 → 5/7 cholangitis, 1/7 noro virus, 1/7 localization not known; [f]4/5 cholangitis, 1/5 urinary tract infection

Tab. 3 Postoperative complications

		Dindo's Grade [36]				
		All (1-5)	1-2	3a/3b	4a/4b	5
Surgery alone (Arm-A)		32	17	9	4	2
As treated (n=37)	Resection (n=26)	23	12	6	4	1
	Exploration (n=11)	9	5	3	0	1
CRT and surgery (Arm-B)		22	16	6	0	0
As treated (n=29)	Resection (n=16)	14	9	5	0	0
	Exploration (n=9)	8	7	1	0	0
	(no surgery n=4)	-	-	-	-	-
Total (n=66)	Resection (n=42)	37	21	11	4	1
	Resection (n=42)	17	12	4	0	1
	no surgery (n=4)		-	-	-	-

Abbreviations: CRT, chemoradiotherapy

26 patients had surgery at a median of day 45 (range 41-59) after completion of chemoradiation, i.e. day 105 (median) after randomization (range 91-119). 19/33 patients had tumor resection and 4 patients had extended surgery to achieve R0-resection. Ten of 33 patients had an explorative laparotomy. Four patients had no surgery due to progressive disease. Resection rates between the arms were not different (P=0.31). In Arm-B, 3/4 patients without chemoradiation had tumor resection; one had liver metastases at exploration.

Of importance, patients in Arm-B did not have elevated rates of high-grade post-operative complications (Dindo´s classification, Tab. 3). [36]

One patient died as the result of an intraoperative myocardial infarction after tumor resection (Arm-A) and one patient died due to sepsis possibly due to cholangitis after explorative laparotomy (Arm-A). One patient had insufficiency of the pancreatico-jejunal anastomosis followed by multi-organ dysfunction (grade 4b; Arm-A, none in Arm-B). The most severe post-operative complications after chemoradiation were grade 3b (intervention under general anesthesia) due to intraabdominal abscess/ fluid retention (n=4) or insufficiency of the choledochojejunal anastomosis (n=1). In Arm-A 10/23 patients had adjuvant chemotherapy and in Arm-B 7/19 patients.

Outcome

The median follow-up for all living patients was 61 months (range 37-79). There were 29 deaths in Arm-A and 31 deaths in Arm-B and local relapse was higher in Arm-A versus B 11/23 versus 8/19. At intention-to-treat analysis mOS between the two arms was not significantly different for all patients irrespective of resection status (Arm-A, 14.4 months; Arm-B 17.4 months; P=0.96; Fig. 2A).

After resection mOS was 18.9 months (Arm-A) versus 25.0 months (Arm-B) (P= 0.79; intention-to-treat analysis). The recurrence pattern of patients after complete tumor resection showed slightly less local recurrences as first site of progression after chemoradiation (local recurrence 6/23 versus 3/19, distant metastases 6/23 versus 10/19, both 5/23 versus 2/19, unknown or no tumor recurrence 6/23 versus 4/19).

The mDFS was 12.1 versus 13.7 months (Arm-A versus Arm-B; P=0.83). Time to progression measured 8,7 versus 8,4 months (Arm-A versus Arm-B; P=0,95; Fig. 2B).

Pathohistological diagnosis of pancreatic adenocarcinoma at biopsy was confirmed in 42/44 resection specimens. 1 distal choledochal adenocarcinoma [Arm-B] and 1 duodenal adenocarcinoma [Arm-A] were excluded from all analyses. R0-resections were achieved in 16/33 versus 17/33 patients (Arm-A versus Arm-B;0.81), and mOS was 18.9 months (Arm-A) versus 25.9 months

Tab. 4 Pathological Staging

		Surgery alone (Arm A)	CRT and surgery (Arm B)
Characteristic	Variable	N=23	N=19
Pathological T category*	(y)pT1	0	2
	(y)pT2	2	2
	(y)pT3	20	15
	(y)pT4	1	0
Pathological N category*	(y)pN0	10	13
	(y)pN1	13	6
Pathological M category*	(y)pM0	21	17
	(y)pM1	2	2
Pathological UICC stage*	(y)pI	1	4
	(y)pII	19	13
	(y)pIII	1	0
	(y)pIV	2	2
Grading	G1	0	0
	G2	11	9
	G3	10	8
	G4	2	1
	Not specified	0	1
Resection margin	R0	16	17
	R1	7	2

Abbreviations: CRT, chemoradiotherapy
*According to UICC 2002

(Arm-B) (P=0.75) (Fig. 2C). Nodal status was (y)pN0 in 10/33 and 13/33 patients in Arm-A and Arm-B, respectively (P=0.44). (y)pN0-status resulted in significantly longer mOS in Arm-A (Fig. 2D). 4 patients had pathological proven distant metastases resected (Arm-A n=2 [lymph node, duodenum]; Arm-B n=2 [lymph node]). Pathological results for resected patients are listed in Tab. 4. Post-resection tumor regression grading in Arm-B did not correlate with OS and was: 10-90% in 8/19, <10% regression in 4/19, >90% in 3/19.

Discussion

The planning of this trial was started in 1999 with activation in 2003 before neoadjuvant treatment had become standard for other diseases, e.g. rectal carcinoma, and therefore had to overcome resistance by physicians and patients likewise against the idea of neoadjuvant treatment as such. Additionally, competing adjuvant trials (CONKO-001[15], ESPAC-3[17]) reduced participation. Another issue was histological or cytological proof of disease before randomization. To overcome this obstacle to recruitment, the protocol allowed randomization after histological proof during explorative laparotomy. Nevertheless, recruitment speed was significantly hampered by these factors. However, to our knowledge this remains the first planned and evaluated multicenter RCT comparing immediate surgery with surgery after neoadjuvant therapy in resectable pancreatic cancer, defined as vascular abutment of less than 180°. But due to low patient numbers this is a negative trial and no clear conclusion can be drawn from underpowered data and whether there is an advantage for one therapy strategy or not.

The following issues of a randomized controlled trial for resectable pancreatic cancer have to be addressed in future trial protocols: working in interdisciplinary teams, predicting resectabilitiy, surgical staging prior to preoperative therapy, definition of vascular resection aims, definition of criteria for cancelling tumor resection during explorative laparotomy and adjuvant chemotherapy. With the nationwide launching of interdisciplinary tumor boards during the last 5 years patients can be screened during theese sessions and it does not longer depend on which specialist the patient contacts first during the course of finding the diagnosis of pancreatic cancer. One of the main problems remains how to predict resectable tumor stage at diagnosis as 20% of tumors without contact to the peripancreatic vessels at diagnosis were not resected with and without neoadjuvant chemoradiation (data not shown). Clearly, the new definition of borderline resectable pancreatic cancer is helpful, but has to be evaluated in future trials. One more point of discussion is vascular resection. Because it was left up to the surgeon to perform a vascular resection to achieve R0-resection this might cause a bias, which is difficult to figure out. A further point of discussion is the different judgement between centers with reference to cancelling surgery, as only one center did abandon resection of the tumor after detection of distant lymph node metastasis (2 pts.) or did not proceed to surgery when progression (locally, distant, clinically) at restaging after chemoradiation was seen (Fig. 1). Another bias might be caused by

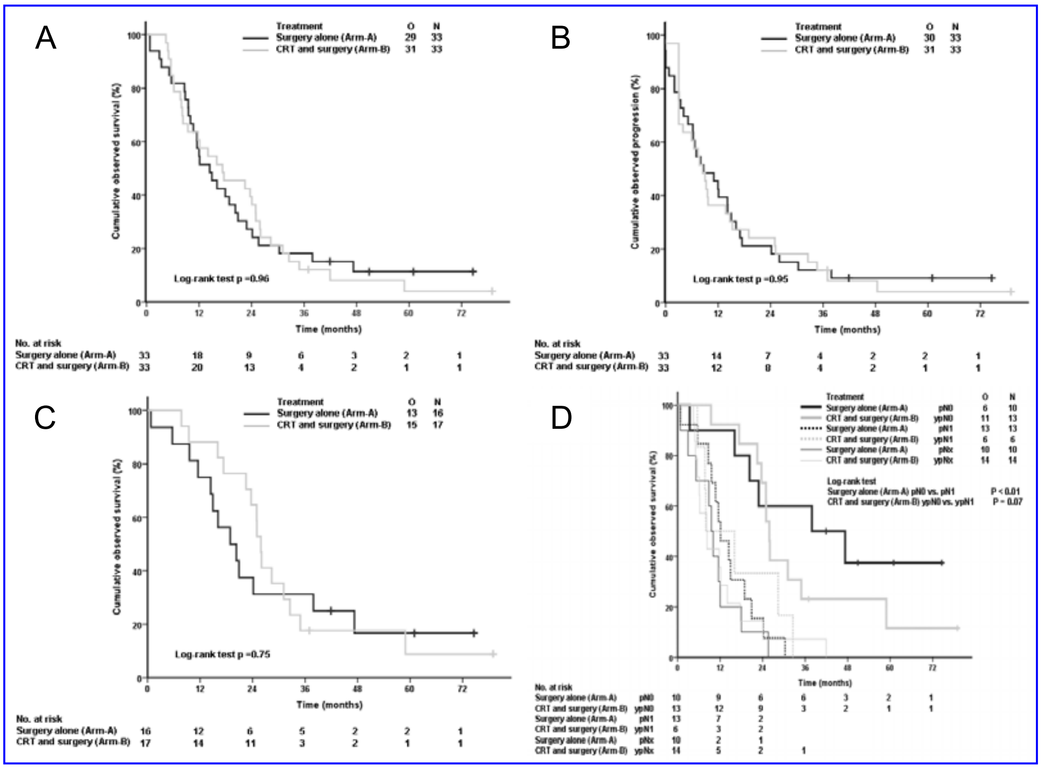

Fig.2 ◀ Kaplan Meier curves (intention to treat analysis) for A Overall survival, B Time to progression, C Overall survival after R0-resection and D Overall survival according to (y)pN-status. CRT, chemoradiation; O, events (deaths [A, C and D] or progression of disease [B]) observed; N, overall number; pNx no tumor resection [D].

adjuvant chemotherapy. As adjuvant chemotherapy became standard during the conduction of this trial an amendment was added, which suggested adjuvant chemotherapy for all patients. The initial consideration was, that the addition of adjuvant chemotherapy does only marginally influence the results of this trial. Because the biggest impact on survival has resection of the tumor and this trial confirms that only tumor resection does lead to long term survival (i.e. more than 25 months) with or without neoadjuvant treatment. But as the resection rates between the groups do not differ significantly the influence of adjuvant chemotherapy on the result of this trial should increase and hence misguide.

A further problem in conducting this trial was proof of histology. Both patients with diverging histology had pathologically proven “adenocarcinoma”, and radiological results prompted the diagnosis of adenocarcinoma of the pancreatic head. But at surgery the primary tumor evolved as distal choledochus cancer and duodenal cancer, respectively. Both patients were better not included into the study if they had properly diagnosed with tumor of the papilla of vateri. Not all centers had excellent interventional radiologists experienced in core needle biopsy of the head of the pancreas. Therefore two centers randomized patients during explorative laparotomy after establishing histological diagnosis on fresh frozen biopsies. The initially mandatory laparoscopy was reclassified as optional due to objections of potential trial participants in an amendment in 2004. Because discrete peritoneal carcinomatosis or subserous small liver metastases can escape detection by CT-scan and will only be detected by staging laparoscopy or –tomy, patient randomization was stratified for laparoscopy, but unfortunately not surgical staging therefore not including explorative laparotomy. Altogether, surgical staging was conducted only in 54% of all patients and mOS for patients in Arm-B with prior surgical staging outmatched mOS for patients without surgical staging (data not shown), confirming an observation already made by others, too.[21] Therefore prior surgical staging should be considered further trials on preoperative treatment strategies.

Actual survival was significantly higher in both arms compared to the historic controls used for statistical planning of this trial. The closest possible comparison of this trial is with adjuvant treatment, especially with the CONKO-001 trial conducted in the same population and with an observation arm.[15, 37] However, the fundamental difference between the here reported trial and adjuvant treatment is, that the latter only includes patients after resection and pathological staging, whereas in this study 24/68 of the patients (35%) had reasons preventing curative resection despite of suggested resectability at staging. Median overall survival in the CONKO-001 trial was 20.2 and 22.1 months (control versus adjuvant gemcitabine, P=0.06). This compares well with the mOS of patients with resections in this trial (18 and 25 months; Arm-A versus Arm-B). In CONKO-001, resection margin status was a negative prognostic marker in the observation arm (mOS 20.8 and 14.1 months R0 versus R1). Recent reports about the lack of prognostic significance of margins might be related to frequent underreporting of R1-status because series with high R1-resection rates correlated with the highest prognostic value of margin status. Therefore, higher R0-resection rates after neoadjuvant treatment are expected to impact on survival.[8, 10, 11, 23, 24]

Neoadjuvant treatment did not show an effect in this strongly underpowered trial due to underrecruitment, but on the other hand was a suitable instrument for selecting patients for surgery. Patients with initially unknown distant metastases might be unmasked by preoperative therapy and hence spared from surgery.[22] In this trial, all patients with neoadjuvant treatment survived at least 3 months whereas after primary surgery 3/34 patients died within this timeframe. Additionally, less severe complications were seen after chemoradiation therapy, probably due to induction of fibrosis, which improves the suitability of pancreatic tissue for anastomosis. A recent meta-analysis also found similar perioperative morbidity with and without neoadjuvant treatment.[24]

Toxicity of chemoradiotherapy was well manageable in this trial, because no patient receiving chemoradiation had an interruption of radiotherapy and only 3/30 patients had delay, reduction or omission of the last dose of chemotherapy. Due to education of the participating doctors and patients, the well. The well known risk of biliary stent dysfunction was managed by prompt stent replacement, but was the most frequent reason for severe adverse events. On the other hand there is a great debate on the minimum case load for pancreatic cancer surgery, because of the high morbidity and mortality of the procedure if it is not done by experienced surgeons. Surely the same issue applies to the chemoradiation therapy, which shouldn`t be conducted by health care institutions not accustomed to this difficult organ and without the prompt support from other medical disciplines as endoscopy or hematology. Gemcitabine-based chemoradiation[14, 38] is increasingly accepted as an alternative standard to fluoropyrimidine-based chemoradiation[39] and was recently shown to be superior to chemotherapy only in locally advanced pancreatic cancer.[40] Hematologic toxicity of gemcitabine-based CRT is directly related to radiotherapy volume and therefore volumes were strictly limited.[38, 41, 42] Additionally, consequent supportive therapy may explain the improved tolerability of treatment in this trial compared to others avoiding loss of weight which was described to be a negative prognostic factor after neoadjuvant chemoradiotherapy.[43] The patients in this trial were treated

with 3D-conformal plans which have recently been shown to be equally effective and not significantly more toxic as IMRT plans in the neoadjuvant setting.[44]

Furthermore, predicting resectability based on CT-scans was difficult and proceeding to resection hereby defining the golden goal of R0-resection remains controversely. Thus, the CONKO-007 (NCT01827553) trial will study the role of chemoradiation in borderline resectable and nonresectable pancreatic cancer. A panel of highly experienced surgeons will review all CT-scans before registering to the trial and at restaging and give their statement about resectability. With the experience of such a trial the criteria of R0 resectability will be evaluated and adjusted and after knowing the significance of chemoradiation for locally advanced and borderline resectable pancreatic cancer the next step might be a phase II trial testing the R0 resectability with neoadjuvant therapy.

Furthermore molecular markers to predict locally predominant growth are emerging, and we should aim to personalize management decisions with regard to neoadjuvant treatment intensification on the basis of these biological characteristics.[45, 46]

In conclusion, we here present the results of an RCT implicating the strategy of multimodal therapy for (borderline) resectable pancreatic cancer which was visionary at the time of planning and conduction of the trial; it has been nearly 15 years ahead of time before this approach was again implemented into prospective trials in Europe. In the meantime the conditions for conduction of interdisciplinary trials have improved much due to governmental regulations and nationwide implementation of certified cancer centers with interdisciplinary tumor boards and the requestion of qualified study nurses supporting the medical study team in the long term. The improvement of interdisciplinary study structures and the lack of better therapies evolving in the meantime led to boycotting this trial to copying the treatment strategy of neoadjuvant chemoradiation with starting a nearly identical study protocol in August 2013 (NCT01900327). Prediction of resectability preoperatively is still an unresolved problem and the long-term results of treatment for pancreatic cancer are still frustrating even after complete resection of the tumor, therefore at the moment we don`t have a better choice but investigating new treatment strategies suitable for as many patients with pancreatic cancer as possible.

Corresponding address

Dr. H. Golcher
Department of Surgery, University Hospital Erlangen
Krankenhausstr. 12, 91054 Erlangen
Germany
henriette.golcher@uk-erlangen.de

Acknowledgments. We are grateful to the patients who participated in this study. We thank all of the investigators who participated in this study: N. Christen, S. Kißenkötter, H. Lauer, V. Lück, Krankenhaus Dresden-Friedrichstadt, Dresden; B. Adamietz, A. Agaimy, U. Baum, R. Croner, A. Dimmler, M. Geiger, S. Herold, R. Janka, A. Kergaßner, P. Klein, S. Krüger, M. Lindenberg, W. Melzner, T. Papadopoulos, J. Pelz, A. Schlabrakowski, U. von Linden, C. Weiß, M. Zeilinger, Universitätsklinikum Erlangen, Erlangen; C. Gog, D. Imhoff, C. Wullstein, Universitätsklinikum Frankfurt, Frankfurt/Main; H. Bockhorn, A. Hildebrand, Krankenhaus Nordwest, Frankfurt/Main; J. Behrbohm, K. Gumpp, J. Hauss, A. Liebmann, Universitätsklinikum Leipzig, Leipzig; H. J. Schlitt, C. Zühlke, Universitätsklinikum Regensburg, Regensburg; T. Horbach, Krankenhaus Schwabach, Schwabach; S. Bischofberger, P. Folie, D. Hausmann, D. Köberle, J. Lange, C. Meyenberger, I. Neuweiler, G. Ries, M. Zünd, Kantonsspital St. Gallen, St. Gallen.

Funding. This work was supported by Deutsche Krebshilfe (70–3046-Ho 2 to W.H.) and Verein zur Förderung des Tumorzentrums der Universität Erlangen-Nürnberg e. V.

Compliance with ethical guidelines

Conflict of interest. R. Fietkau received honoraria from Eli Lilly and Company (“Lilly”), Fresenius SE & Co. KGaA, and F. Hoffmann-La Roche Ltd. H. Golcher, T.B. Brunner, H. Witzigmann, L. Marti, W.-O. Bechstein, C. Bruns, H. Jungnickel, S. Schreiber, G.G. Grabenbauer, T. Meyer, S. Merkel, and W. Hohenberger state that there are no conflicts of interest.

The study has been presented in part during the following meetings by oral presentation (op) or discussed poster (dp): World Congress GI-Cancer 2012, Barcelona (dp); Annual Meeting ESTRO 2012, Barcelona (op); 21. Chirurgisches Friedrichstädter Symposion May 2012, Dresden (op); Deutscher Chirurgenkongress 2012, Berlin (op); Deutscher Krebskongress 2012, Berlin (dp); Viszeralmedizin 2011, Leipzig (op); Viszeralmedizin 2010, Stuttgart (op).

References

1. Siegel R, Naishadham D, Jemal A (2012) Cancer statistics, 2012. CA Cancer J Clin 62:10-29
2. Cunningham D, Atkin W, Lenz HJ, et al (2010) Colorectal cancer. Lancet 375:1030-47
3. Heald RJ, Moran BJ, Ryall RD, et al (1998) Rectal cancer: the Basingstoke experience of total mesorectal excision, 1978-1997. Arch Surg 133:894-9
4. Glimelius B, Pahlman L, Cervantes A (2010) Rectal cancer: ESMO Clinical Practice Guidelines for diagnosis, treatment and follow-up. Ann Oncol 21 Suppl 5:v82-6
5. Sauer R, Becker H, Hohenberger W, et al (2004) Preoperative versus Postoperative Chemoradiotherapy for Rectal Cancer. N Engl J Med 351:1731-40
6. Rodel C, Liersch T, Hermann RM, et al (2007) Multicenter phase II trial of chemoradiation with oxaliplatin for rectal cancer. J Clin Oncol 25:110-7
7. Quirke P, Durdey P, Dixon MF, et al (1986) Local recurrence of rectal adenocarcinoma due to inadequate surgical resection. Histopathological study of lateral tumour spread and surgical excision. Lancet 2:996-9
8. Esposito I, Kleeff J, Bergmann F, et al (2008) Most pancreatic cancer resections are R1 resections. Ann Surg Oncol 15:1651-60
9. Hidalgo M (2010) Pancreatic cancer. N Engl J Med 362:1605-17
10. Menon KV, Gomez D, Smith AM, et al (2009) Impact of margin status on survival following pancreatoduodenectomy for cancer: the Leeds Pathology Protocol (LEEPP). HPB (Oxford) 11:18-24
11. Zhang Y, Frampton AE, Cohen P, et al (2012) Tumor infiltration in the medial resection margin predicts survival after pancreaticoduodenectomy for pancreatic ductal adenocarcinoma. J Gastrointest Surg 16:1875-82
12. Witkowski ER, Smith JK, Tseng JF (2012) Outcomes following resection of pancreatic cancer. J Surg Oncol 107:97-103
13. Simons JP, Shah SA, Ng SC, et al (2009) National complication rates after pancreatectomy: beyond mere mortality. J Gastrointest Surg 13:1798-805
14. Neoptolemos JP, Stocken DD, Friess H, et al (2004) A randomized trial of chemoradiotherapy and chemotherapy after resection of pancreatic cancer. N Engl J Med 350:1200-10
15. Oettle H, Post S, Neuhaus P, et al (2007) Adjuvant chemotherapy with gemcitabine vs observation in patients undergoing curative-intent resection of pancreatic cancer: a randomized controlled trial. JAMA 297:267-77
16. Regine WF, Winter KA, Abrams RA, et al (2008) Fluorouracil vs gemcitabine chemotherapy before and after fluorouracil-based chemoradiation following resection of pancreatic adenocarcinoma: a randomized controlled trial. JAMA 299:1019-26
17. Neoptolemos JP, Stocken DD, Bassi C, et al (2010) Adjuvant chemotherapy with fluorouracil plus folinic acid vs gemcitabine following pancreatic cancer resection: a randomized controlled trial. JAMA 304:1073-81
18. Adler G, Seufferlein T, Bischoff SC, et al (2007) [S3-Guidelines "Exocrine pancreatic cancer" 2007]. Z Gastroenterol 45:487-523
19. Papalezova KT, Tyler DS, Blazer DG, 3rd, et al (2012) Does preoperative therapy optimize outcomes in patients with resectable pancreatic cancer? J Surg Oncol 106:111-8
20. Wayne JD, Abdalla EK, Wolff RA, et al (2002) Localized adenocarcinoma of the pancreas: the rationale for preoperative chemoradiation. Oncologist 7:34-45
21. Snady H, Bruckner H, Cooperman A, et al (2000) Survival Advantage of Combined Chemoradiotherapy Compared with Resection as the Initial Treatment of Patients with Regional Pancreatic Carcinoma. An Outcomes Trial. Cancer 89:314–27
22. Evans DB, Varadhachary GR, Crane CH, et al (2008) Preoperative gemcitabine-based chemoradiation for patients with resectable

adenocarcinoma of the pancreatic head. J Clin Oncol 26:3496-502
23. Golcher H, Brunner T, Grabenbauer G, et al (2008) Preoperative chemoradiation in adenocarcinoma of the pancreas. A single centre experience advocating a new treatment strategy. Eur J Surg Oncol 34:756-64
24. Gillen S, Schuster T, Meyer Zum Buschenfelde C, et al (2010) Preoperative/neoadjuvant therapy in pancreatic cancer: a systematic review and meta-analysis of response and resection percentages. PLoS Med 7:e1000267
25. Brunner TB, Grabenbauer GG, Meyer T, et al (2007) Primary resection versus neoadjuvant chemoradiation followed by resection for locally resectable or potentially resectable pancreatic carcinoma without distant metastasis. A multi-centre prospectively randomised phase II-study of the Interdisciplinary Working Group Gastrointestinal Tumours (AIO, ARO, and CAO). BMC Cancer 7:41
26. Lu DS, Reber HA, Krasny RM, et al (1997) Local staging of pancreatic cancer: criteria for unresectability of major vessels as revealed by pancreatic-phase, thin-section helical CT. Am J Roentgenol 168:1439-43
27. Brunner TB, Grabenbauer GG, Kastl S, et al (2000) Preoperative Chemoradiation in Locally Advanced Pancreatic Carcinoma: A Phase II Study. Onkologie 23:436-42
28. Wilkowski R, Thoma M, Schauer R, et al (2004) Effect of chemoradiotherapy with gemcitabine and cisplatin on locoregional control in patients with primary inoperable pancreatic cancer. World J Surg 10:1011-8
29. Fokas E, Eccles C, Patel N, et al (2013) Comparison of four target volume definitions for pancreatic cancer. Guidelines for treatment of the lymphatics and the primary tumor. Strahlenther Onkol 189:407-16
30. Sobin L, Wittekind C, eds. International Union Against Cancer (UICC). TNM classification of malignant tumors. 5th edition ed. New York: John Wiley & Sons, Inc.; 1997.
31. Sobin LH, Wittekind C, eds. TNM classification of malignant tumours. 6th ed. New York: Wiley & Sons; 2002.
32. Evans DB, Rich TA, Byrd DR, et al (1992) Preoperative chemoradiation and pancreaticoduodenectomy for adenocarcinoma of the pancreas. Arch Surg 127:1335-9
33. Therasse P, Arbuck SG, Eisenhauer EA, et al (2000) New guidelines to evaluate the response to treatment in solid tumors. European Organization for Research and Treatment of Cancer, National Cancer Institute of the United States, National Cancer Institute of Canada. J Natl Cancer Inst 92:205-16
34. National Cancer Institute Common Toxicity Criteria, Version 2.0: Cancer Therapy Evaluation Program., 1998. (Accessed 1.9.2014 at http://ctep.cancer.gov/protocolDevelopment/electronic_applications/docs/ctcv20_4-30-992.pdf.)
35. Cox JD, Stetz J, Pajak TF (1995) Toxicity criteria of the Radiation Therapy Oncology Group (RTOG) and the European Organization for Research and Treatment of Cancer (EORTC). Int J Radiat Oncol Biol Phys 31:1341-6
36. Dindo D, Demartines N, Clavien PA (2004) Classification of surgical complications: a new proposal with evaluation in a cohort of 6336 patients and results of a survey. Ann Surg 240:205-13
37. Oettle H, Neuhaus P, Hochhaus A, et al (2013) Adjuvant chemotherapy with gemcitabine and long-term outcomes among patients with resected pancreatic cancer: the CONKO-001 randomized trial. JAMA 310:1473-81
38. **Zhu CP**, **Shi J**, Chen YX, et al (2011) Gemcitabine in the chemoradiotherapy for locally advanced pancreatic cancer: a meta-analysis. Radiother Oncol 99:108-13
39. Chauffert B, Mornex F, Bonnetain F, et al (2008) Phase III trial comparing intensive induction chemoradiotherapy (60 Gy, infusional 5-FU and intermittent cisplatin) followed by maintenance gemcitabine with gemcitabine alone for locally advanced unresectable pancreatic cancer. Definitive results of the 2000-01 FFCD/SFRO study. Ann Oncol 19:1592-9
40. Loehrer PJ, Sr., Feng Y, Cardenes H, et al (2011) Gemcitabine alone versus gemcitabine plus radiotherapy in patients with locally advanced pancreatic cancer: an Eastern Cooperative Oncology Group trial. J Clin Oncol 29:4105-12
41. Brunner TB, Merkel S, Grabenbauer GG, et al (2005) Definition of elective lymphatic target volume in ductal carcinoma of the pancreatic head based on histopathologic analysis. Int J Radiat Oncol Biol Phys 62:1021-9
42. Crane CH, Wolff RA, Abbruzzese JL, et al (2001) Combining gemcitabine with radiation in pancreatic cancer: understanding important variables influencing the therapeutic index. Semin Oncol 28:25-33
43. Naumann P, Habermehl D, Welzel T, et al (2013) Outcome after neoadjuvant chemoradiation and correlation with nutritional status in patients with locally advanced pancreatic cancer. Strahlenther Onkol 189:745-52
44. Combs SE, Habermehl D, Kessel K, et al (2013) Intensity modulated radiotherapy as neoadjuvant chemoradiation for the treatment of patients with locally advanced pancreatic cancer. Outcome analysis and comparison with a 3D-treated patient cohort. Strahlenther Onkol 189:738-44
45. Iacobuzio-Donahue CA, Fu B, Yachida S, et al (2009) DPC4 gene status of the primary carcinoma correlates with patterns of failure in patients with pancreatic cancer. J Clin Oncol 27:1806-13
46. Crane CH, Varadhachary GR, Yordy JS, et al (2011) Phase II trial of cetuximab, gemcitabine, and oxaliplatin followed by chemoradiation with cetuximab for locally advanced (T4) pancreatic adenocarcinoma: correlation of Smad4(Dpc4) immunostaining with pattern of disease progression. J Clin Oncol 29:3037-43
47. Schulz KF, Altman DG, Moher D (2010) CONSORT 2010 statement: updated guidelines for reporting parallel group randomised trials. BMJ 340:c332

Author names in **bold** designate shared co-first authorship.
